# A Study on the Efficacy and Pharmacological Mechanism of Liposome Complexes Containing STING Agonist and Anti-PD-L1 Nanobody in Inhibiting HCC

**DOI:** 10.3390/ijms26178649

**Published:** 2025-09-05

**Authors:** Xiaoqing Wang, Xing Lu, Chang Liu, Hao Cheng, Xiangshi Tan

**Affiliations:** Department of Chemistry, Fudan University, Shanghai 200433, China; 20110220119@fudan.edu.cn (X.W.);

**Keywords:** STING agonist, cGAMP, anti-PD-L1 nanobody, nanobody, hepatocellular carcinoma (HCC)

## Abstract

The low immunogenicity and immune escape are bottlenecks for effective hepatocellular carcinoma (HCC) immunotherapy. We prepared and characterized a dual-target liposome complex, XA5508, by encapsulating the STING agonist cGAMP in liposomes and conjugating an anti-PD-L1 nanobody to the liposome surface. The anti-tumor effect and pharmacological mechanism of XA5508 were investigated using an in situ HCC mouse model. XA5508 can effectively inhibit in situ HCC with the characteristics of tumor-targeted delivery and sustained release of STING agonist cGAMP. The pharmacological mechanism study indicates that XA5508 activates the STING signaling pathway, increases the cytotoxicity of CD8^+^ T cells, reverses the immunosuppressive tumor microenvironment (TME) represented by M2-type macrophages, and transforms cold tumors into hot tumors. On the other hand, cGAMP induces the upregulation of PD-L1 expression in HCC, enhances the response of anti-PD-L1 nanobody (Nb) and the escape blockade of immune checkpoint PD-1/PD-L1. XA5508 shows remarkable anti-tumor effects of STING agonist and anti-PD-L1 nanobody against HCC, providing an innovative strategy for the development of new drugs for HCC.

## 1. Introduction

Hepatocellular carcinoma (HCC), as the most common form of primary liver cancer, is the second leading cause of cancer-related deaths worldwide [1]. The 5-year overall survival rate for HCC is less than 20% [2]. The early treatment for liver cancer includes surgical resection, liver transplantation, and percutaneous ablation. Surgical removal can increase the survival rate, with a 50% to 70% increase within 5 years [3]. However, the risk of tumor recurrence and metastasis remains as high as 70%, and the prognosis is poor [4]. Due to the insidious onset of HCC and the absence of early pathological symptoms, most patients are diagnosed as being in the advanced or middle advanced stage when the disease is discovered, and the treatment options are very limited [5]. Because the liver’s metabolic function accelerates the breakdown of drugs and reduces the local drug concentration, monotherapy is prone to drug resistance [6] and can easily damage normal liver tissue, thereby aggravating liver function impairment. These problems increase the complexity of treatment.

When clinical drugs such as multi-target tyrosine kinase inhibitors (mTKI), sorafenib, and chemotherapy drugs are used to treat advanced HCC, they usually cause severe adverse reactions, and the overall prognosis is not satisfactory [7]. In recent years, the basic and translational research on programmed cell death protein-1 (PD-1) or programmed death ligand 1 (PD-L1) has become a hot topic in the field of HCC research. Monoclonal antibody drugs that block the immune checkpoint PD-1/PD-L1 have achieved significant breakthroughs in the treatment of advanced HCC patients. However, only a very low rate of tumors is sensitive to the immune checkpoint inhibitors due to their heterogenetic nature and low immunogenic tumor microenvironment (TME) [8,9,10]. The combination therapy of PD-L1 inhibitors and anti-angiogenic drugs, especially the regimen of atezolizumab combined with bevacizumab, has been recommended as the preferred treatment option for patients with advanced HCC by major clinical guidelines [11]. It is noteworthy that the immunotherapy combination of PD-1 inhibitor Opdivo (nivolumab) and CTLA-4-targeted antibody Yervoy (ipilimumab) is the first dual-immune-checkpoint-inhibitor regimen approved for the treatment of advanced HCC patients [12]. However, the complex immune-suppressive microenvironment of HCC leads to the escape of immune checkpoint inhibitors through unknown mechanisms [13,14], which remains a major challenge for therapeutic intervention in HCC patients [15]. Thus, enhancing a robust antitumor immune response is essential for successful immunotherapy, especially for those tumors with poorly immunogenic tumors [16].

In recent years, the stimulator of interferon genes (STING) signaling pathway has been identified as a key target for tumor immunotherapy [17,18,19]. Therefore, activating the STING pathway has become a prominent focus in the development of anti-tumor drugs, and several STING agonists have entered the clinical research stage [20,21]. The metalloenzyme cGAS, upon recognizing and binding to dsDNA, catalyzes the synthesis of ATP and GTP to produce the endogenous STING agonist 2′,3′-cGAMP (cGAMP) [22]. As a second messenger, cGAMP binds to STING in the endoplasmic reticulum. Then, STING recruits and activates TBK1 (which binds to TANK and kinase 1). TBK1 phosphorylates IRF3, inducing the production of IFN-I (type I interferon) and a series of inflammatory immune responses [23,24]. The cGAS-STING pathway plays a significant role in innate immunity and host defense. Moreover, the selectively activated STING in tumor cells can stimulate and produce some chemokines and cytokines such as type I IFN, which can recruit tumor-infiltrating lymphocytes and increase the sensitivity of the initially cold tumors to ICB therapy [25]. In addition, activating STING in tumor endothelial cells can disrupt abnormal vascular patterns, thereby increasing drug accumulation within the tumor [26].

At present, research on STING agonists as a treatment strategy for HCC is still ongoing. Some studies have shown that they have significant effects in prolonging survival and enhancing the immune response to tumors. Research indicates that hepatocytes do not express STING, so the pathway cannot be activated, while non-essential cells in the liver can sense cytoplasmic DNA, including Kupffer cells [27]. In liver tumors, Kupffer cells will have a strong response to STING agonists, leading to systemic activation of IFN, induction of tumor cell apoptosis, and activation of autophagy, and jointly initiating the immune response [28]. However, in TME, the innate immune STING pathway is inhibited, which promotes immune escape and tumor progression [28,29]. Meanwhile, STING agonists are generally limited by obstacles such as easy degradation by ENPP1 enzymes [30], difficult to be taken up and transported by cells, and insufficient accumulation in tumors [31,32,33]. These factors make it difficult for STING agonists to achieve effective anti-tumor responses and to minimize potential side effects [32]. Therefore, developing nano-delivery systems to deliver STING agonists to tumor cells is important for selectively activating the STING signal pathway in tumors, enhancing the immune response, and reducing potential side effects [34].

Although the STING pathway provides the necessary inflammatory signals, it also initiates feedback inhibition in the TME by upregulating the expression of PD-L1 in tumor cells [35,36,37,38]. The research report indicates that the number of T cells in patients with HCC is low and severely damaged, which leads to the deterioration of the condition [39]. Therefore, combining the STING agonist with PD-L1 blockade therapy is crucial for achieving effective immunotherapy [16]. Anti-PD-L1 monoclonal antibodies are usually used in cancer immunotherapy, but aPD-L1 usually has problems such as poor diffusion, low tumor penetration ability, and immune-related adverse reactions [40]. Compared with monoclonal antibodies, nanobodies (Nb) without the Fc domain are not only smaller in size, have lower immunogenicity, reduce the occurrence of side effects, improve the therapeutic index, and are easier to prepare with lower costs. In addition, by connecting anti-PD-L1 Nb to nanocarriers, it can actively target specific TME [41], while reducing non-targeted binding to healthy cells, thereby solving some of the main problems of previous monoclonal antibody treatments to a certain extent. Therefore, using anti-PD-L1 Nb to block immune escape is a feasible alternative strategy. Anti-PD-L1 monoclonal antibodies are only sensitive to a small portion of ‘hot’ tumors and are ineffective against ‘cold’ tumors. This requires other approaches (e.g., STING agonists) to activate and enhance the immune response [42]. However, a combination of Nb and STING activation has not been sufficiently studied. To address the instability of ionic complexes under physiological conditions [43] and enhance the efficacy of drug delivery, various alternative strategies have been adopted, including the attachment of ligands to the surface of nanocarriers to achieve active targeting [44]. Stable encapsulation, efficiently delivering drug into the target cells, such as liver cancer cells, and achieving the desired immunotherapy effects remain major challenges [45].

Herein, a dual-target liposome complex XA5508, which encapsulated STING agonist cGAMP and linked murine anti-PD-L1 Nb to the surface of the liposomes, was prepared and characterized. The anti-tumor effect and pharmacological mechanism of the liposome complex XA5508 were investigated using situ HCC mouse models. The XA5508 could slowly release and deliver cGAMP to the TME, which stimulated robust immune responses by tumor-specific activation of STING. Additionally, cGAMP induces the upregulation of PD-L1 expression in the HCC and enhances the response of anti-PD-L1 Nb and the escape blockade of immune checkpoint PD-1/PD-L1 for amplified immunotherapy of HCC. This study presents the preparation and anti-tumor functional analysis of the dual-target liposome complex XA5508 that efficiently delivers immunotherapeutic agents cGAMP to liver tumors, thus activating antitumor immunity and inhibiting immune escape to enable effective immunotherapy of HCC. This provides an innovative strategy for the development of innovative drugs for HCC.

## 2. Results

### 2.1. The Composition and Characteristics of the Liposome Complex XA5508

**Figure 1:**
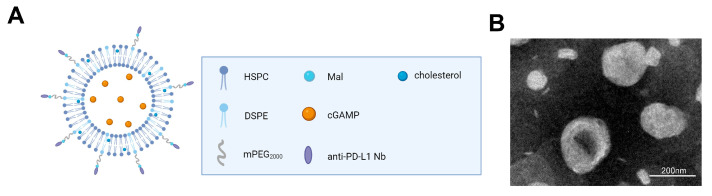
Composition and TEM results of liposome complex XA5508. (**A**) Schematic diagram of the composition of XA5508. (**B**) TEM micrograph results.

The dual-target liposome complex XA5508, which encapsulated STING agonist and linked murine anti-PD-L1 Nb to the surface of the liposome, was designed and prepared. The structure of XA5508 is shown in Figure 1A. According to the transmission electron microscopy imaging, the morphology of XA5508 is spherical (Figure 1B). Dynamic light scattering analysis shows a particle size of approximately 202 nm, with a PDI of less than 0.3, indicating a uniform size distribution (Table 1). The surface charge is negative (−14 mV), which can reduce protein adsorption in the blood and thereby increase its circulation half-life. The encapsulation efficiency (EE) of cGAMP is ~83%, and the loading efficiency (LE) is 8.31%. The concentration of the coupled αPD-L1 VHH is 256.40 mg/mL, and the phospholipid concentration is 12.57 mg/mL. These results indicate that XA5508 has the characteristics of good morphology and efficient encapsulation of cGAMP.

In this study, lipid nanomaterials were used to encapsulate the endogenous STING agonist 2′,3′-cGAMP, ensuring that cGAMP could be delivered to the TME at a sufficient concentration and avoiding degradation in the blood system. On the other hand, what’s important is that the ENPPI enzyme located at the ER can recognize and degrade excessive 2′,3′-cGAMP, thereby preventing the excessive activation of the STING pathway and the occurrence of an immune storm. Furthermore, in order to enhance the efficiency of HCC drug-targeted delivery, an anti-PD-L1 Nb was covalently coupled to the surface of liposomes, enabling them to recognize the overexpressed receptor PD-L1 on tumor cells. This resulted in tumor targeting, allowing the liposomes to achieve active delivery. Ultimately, XA5508 possesses the characteristics of dual targets and multi-functionality, aiming to enhance the efficacy of cancer immunotherapy.

### 2.2. The Liposome Complex XA5508 Targets the TME for Delivery of cGAMP

**Figure 2:**
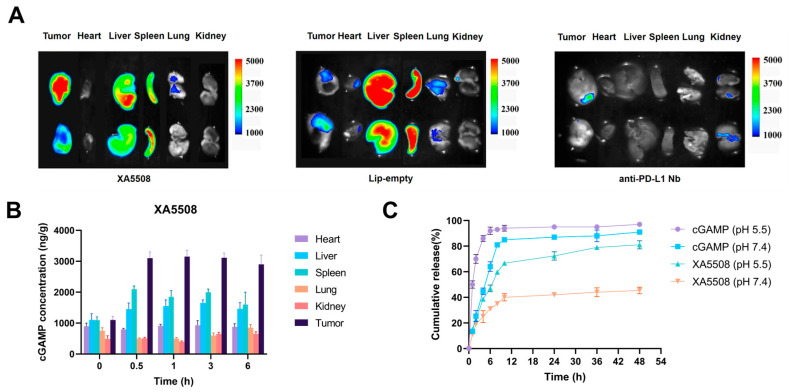
The liposome complex XA5508 can target tumors and deliver cGAMP in a sustained-release manner. (**A**) Fluorescence imaging of the in vitro tissue distribution of DiR-labeled liposomes in Hepal-6 tumor-bearing mice 48 h after injection. (**B**) ELISA detection of the changes in cGAMP content in various tissues at 0, 0.5, 1, 3, and 6 h after injection of the liposome complex XA5508. (**C**) In vitro simulation release test to detect the release curve of cGAMP. *n* = 5, Data are means ± SEM.

To examine whether the liposome complex XA5508 has tumor-targeting properties, we injected mice with subcutaneous tumors with DiR-labeled XA5508, Lip-empty, and anti-PD-L1 Nb via the tail vein. After 48 h, the distribution of the biological tissues through IVIS imaging was observed. As shown in Figure 2A, XA5508 mainly accumulated in the tumor, liver, and spleen, while Lip-empty mainly accumulated in the liver and spleen. Anti-PD-L1 Nb had the weakest fluorescence signal due to its rapid metabolism in the body (Figure S1). These results indicated that the anti-PD-L1 Nb on the surface of the liposome complex can recognize the antigen PD-L1 on the surface of tumor cells, thereby enhancing the internalization of tumor cells by the liposome complex and weakening the RES phagocytosis process. Subsequently, we studied the changes in the content of cGAMP in different tissues at 0, 0.5, 1, 3, and 6 h after tail vein injection of XA5508. The results showed that the content of cGAMP in tumor tissues was obviously higher than that in other tissues, starting from 0.5 h until 6 h (Figure 2B). The release characteristics of cGAMP in the liposome complex XA5508 were verified through in vitro simulated release experiments. As shown in Figure 2C, at the same pH conditions, the release rate of free cGAMP was much higher than that of XA5508. In contrast, XA5508 could slow down the release rate of cGAMP in the simulated neutral environment of blood and accelerate the release in the acidic conditions of the simulated the TME. These results prove that the liposome complex XA5508 has excellent in vivo long circulation and liver tumor targeting effects.

### 2.3. Establishment of Mouse In Situ HCC Model and In Vivo Drug Efficacy

The mouse orthotopic liver cancer model can precisely simulate the morphological, histological and biochemical characteristics of HCC, facilitating the research on clinical treatment strategies and anti-tumor drugs for tumors [46]. Furthermore, as the liver is an important metabolic organ in the human body, its dual blood supply characteristics and close connection with the intestine make the research on HCC more dependent on the use of in situ models compared to other cancer fields [47,48]. Due to the rapid progression of HCC in its middle and late stages, and the varying growth rates of different tumor cell types in the model mice, we need to understand the growth rate and characteristics of the in situ HCC model to determine the treatment window time. In this study, we established an in situ HCC model of Hepal-6 in C57BL/6 mice. The surgical method is referenced in the literature [49], and the surgical operation process is shown in Figure S2A. The tumor growth and survival conditions were checked every 2 days after inoculation. As shown in Figure S2B, the average tumor volume was 152 mm^3^ on the 7th day after inoculation. The average tumor volume was 933 mm^3^ on the 21st day after inoculation. As shown in Figure S2C, the median survival time of Hepa1-6 in the in situ HCC mice were 22 days. Therefore, it can be concluded that it is advisable to end the experiment within 22 days after inoculation of Hepal-6 in situ HCC mice.

**Figure 3:**
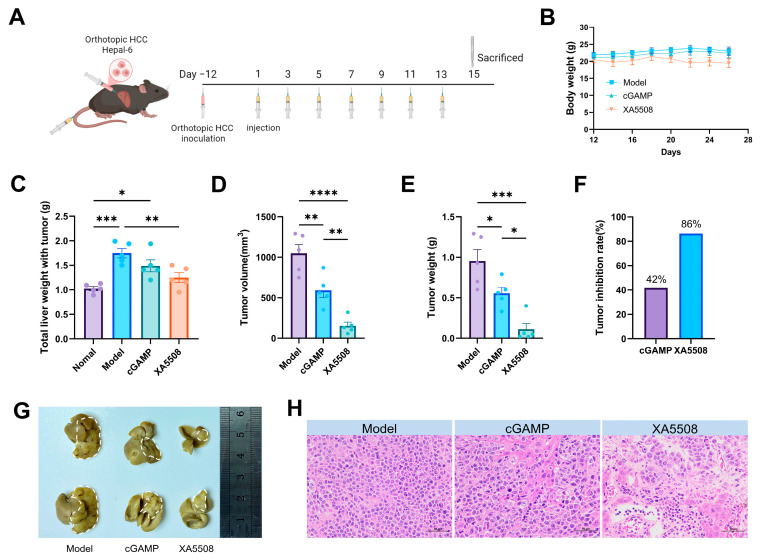
Evaluate the therapeutic effect of liposome complex XA5508 on Hepal-6 in situ liver cancer. (**A**) Schematic diagram of the treatment cycle, (**B**) Body weight, (**C**) Total liver weight with tumor, (**D**) Tumor volume, (**E**) Tumor weight, (**F**) Tumor inhibition rate, (**G**) Representative tumor photos (The white dotted frame in the figure represents the tumor), (**H**) HE staining. Data from one representative experiment out of three total performed. *n* = 5, Data are means ± SEM. One-way ANOVA with Tukey’s HSD significant test was used for pairwise comparisons. *, *p* < 0.05, **, *p* < 0.01, ***, *p* < 0.001, ****, *p* < 0.0001.

Based on the above experiments, the experimental period was designed. Twelve days after the establishment of the Hepal-6 mouse in situ HCC model, the successfully modeled mice were divided into 3 groups: the Model group, the control group (cGAMP), and the XA5508 group. The dosage was calculated based on the included cGAMP and was administered at 10 mg/kg cGAMP. The injection was given every 2 days, for a total of 7 times. During the treatment period, body weight changes were monitored, as shown in Figure 3A. After the treatment, we evaluated the efficacy of XA5508 on the Hepal-6 in situ HCC model of mice and collected blood, liver, spleen, and tumors for statistical analysis. In terms of body weight, XA5508 caused a slight decrease in body weight (Figure 3B). Comparing the total liver weight with tumor of model group, the XA5508 group had the lowest, and the tumor volume and tumor weight trends in the XA5508 group were consistent (Figure 3C–E). Based on the tumor weight, the tumor suppression rate of each group was calculated (Figure 3F), and it was found that the XA5508 group had a tumor suppression rate of 86%, while the control group cGAMP group had a tumor suppression rate of 42%. All the independent repeated experiments showed a similar trend (Figure S3). Combined with the representative tumor images (Figure 3G), it was concluded that XA5508 significantly inhibited the growth of Hepal-6 in situ liver cancer. The HE pathological results of the tumor tissue (Figure 3H) revealed that the tumor cells in the XA5508 group were loosely arranged and had a decreased density, with apoptotic cells visible within the tumor and a large amount of vacuolar accumulation. These data indicate that XA5508 has a good therapeutic effect on in situ HCC.

### 2.4. Safety Testing of Liposome Complex XA5508

To assess whether the systemic administration of the liposome complex XA5508 has adverse effects on circulating hematopoietic cells, we collected blood samples from the animals that received systemic injection for hematological examination. As can be seen from Table 2, the model+XA5508 group showed an increase in the number of white blood cells and neutrophils. Studies have shown that cancer itself and its related treatments can put cancer patients in an intrinsic inflammatory state [50]. This is because the immune regulatory factors produced by tumors, such as granulocyte colony-stimulating factor (G-CSF) lead to an increase in neutrophils [50]. Drug intervention treatment can lead to the secretion of more cytokines, thereby hindering the survival of cancer cells and causing tumor apoptosis [51]. At the same time, the inflammatory response caused some adverse reactions, which might be the reason for the slight weight loss after XA5508 treatment. However, we suspect that this is short-term, as the blood routine values of healthy mice one week after a single administration of XA5508 decreased compared with the control+XA5508 group (24 h) (Supplementary Table S1). These results suggest that XA5508 may stimulate the normal immune system but will not have adverse effects on healthy mice. In terms of red blood cells, hemoglobin and platelets indicators, the results of the XA5508 group were within the normal range. Additionally, in liver, kidney, and heart function indicators, the XA5508 group showed no obvious abnormalities, proving that the safety of XA5508 is good (Table 3). In addition, we also conducted examinations on the non-tumor tissues of the liver (Figure S4), and the results showed that XA5508 did not cause significant damage to the normal liver cells.

### 2.5. The Mechanism of Action of Anti-Tumor Immune Response

**Figure 4:**
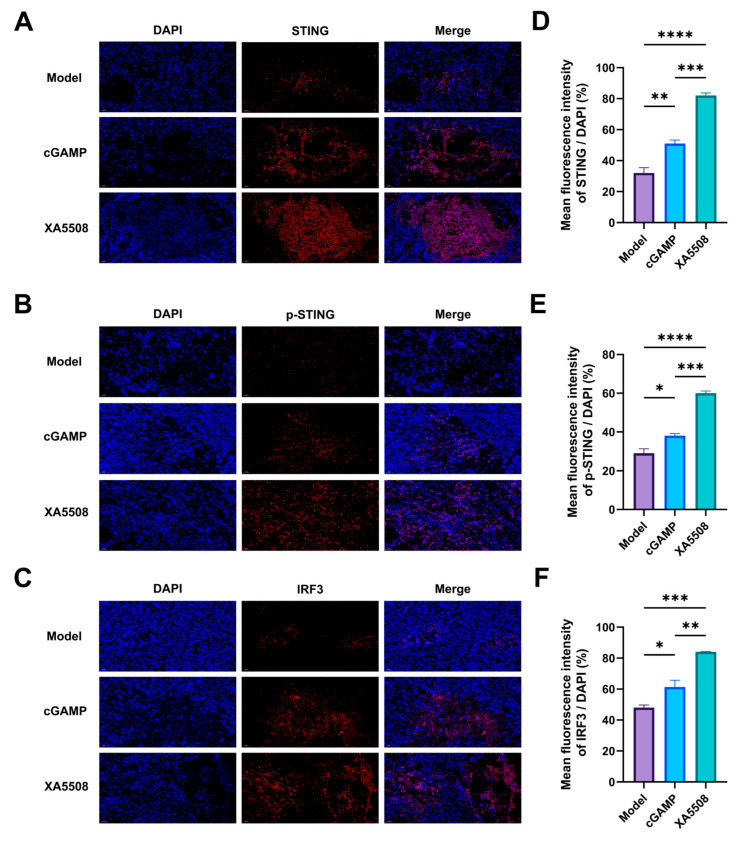
The liposome complex XA5508 activates the STING-IRF3 pathway. Immunofluorescence was used to detect the expression of STING protein (**A**), p-STING protein (**B**), and IRF3 protein (**C**) in each group of tumor tissues from Hepal-6 (*n =* 3). The fluorescence ratios of STING/DAPI (**D**), p-STING/DAPI (**E**), and IRF3/DAPI (**F**) were also measured. Data are means ± SEM. One-way ANOVA with Tukey’s HSD significant test was used for pairwise comparisons. *, *p* < 0.05, **, *p* < 0.01, ***, *p* < 0.001, ****, *p* < 0.0001.

After being recognized and targeted to tumor cells by anti-PD-L1 Nb, the liposomes enter the cells through endocytosis and release their contents, cGAMP. To verify the effective release of cGAMP, we next evaluated whether the liposome complex XA5508 could effectively activate the STING signaling pathway (Figure 4A–C). Through quantitative immunofluorescence analysis of tumor tissues, it was found that compared with the Model group and the cGAMP group, the liposome complex XA5508 significantly increased the expression levels of STING, p-STING, and IRF3 proteins (Figure 4D–F), demonstrating the activation of the cGAMP-STING-IRF3 signaling.

**Figure 5:**
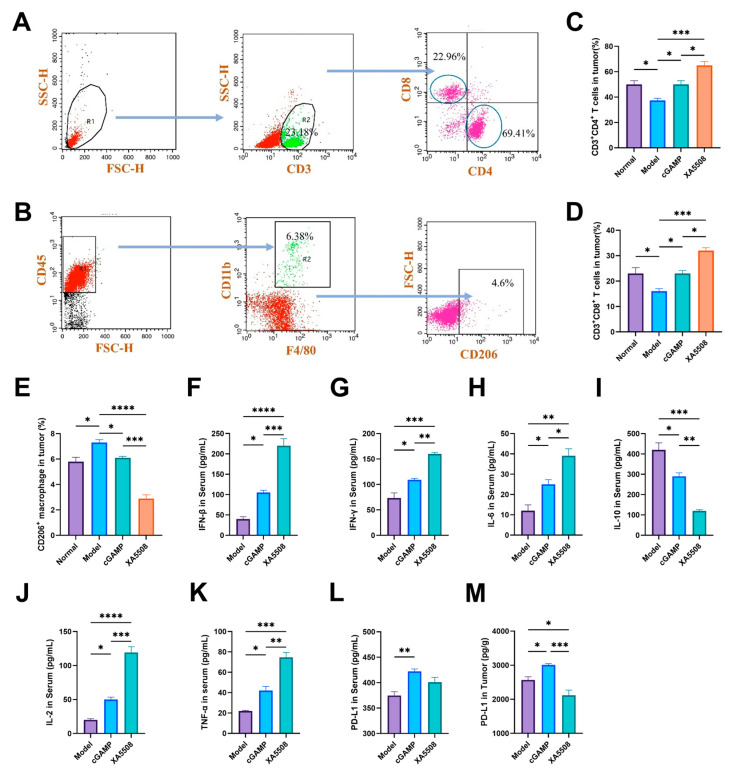
Liposome complex XA5508 reverses the immunosuppressive microenvironment and blocks immune escape. (**A**,**B**) Representative classification strategy for tumor tissue detection markers. (**C**) CD4^+^ T cells in the tumor. (**D**) CD8^+^ T cells in the tumor. (**E**) M2-TAM in the tumor. Serum samples from each group were collected for detection of (**F**) IFN-β, (**G**) IFN-γ, (**H**) IL-6, (**I**) IL-10, (**J**) IL-2, (**K**) TNFα, (**L**,**M**) Expression levels of PD-L1 in serum and tumor of Hepal-6 tumor-bearing mice treated with cGAMP for 7 times. (Data from one representative experiment out of three total performed. *n* = 3, Data are means ± SEM. One-way ANOVA with Tukey’s HSD significant test were used for pairwise comparisons. *, *p* < 0.05, **, *p* < 0.01, ***, *p* < 0.001, ****, *p* < 0.0001).

A large number of studies have shown that the activation of the STING signaling pathway leads to the activation and increase of downstream immune cells and immune factors. However, in HCC, the presence of a large number of M2-type tumor-associated macrophages and the inhibition of dendritic cell (DC) maturation form an immunosuppressive TME with immune tolerance [52]. And the immunosuppressive TME is the main reason for the failure of cancer immunotherapy [53,54]. Therefore, we investigated whether XA5508 could regulate the immunosuppressive TME to restore the efficacy of immunotherapy in HCC. By classifying the immune cells in the TME, we calculated the proportions of tumor-infiltrating CD4^+^ T cells and CD8^+^ T cells and M2-type macrophages in each group (Figure 5A,B). From Figure 5C,D, it can be seen that XA5508 significantly increased the number of tumor-infiltrating CD4^+^ T cells and CD8^+^ T cells. Compared with the model group, the proportion of CD8^+^ T cells increased nearly twice. At the same time, the detection of M2-type macrophage markers revealed that XA5508 could significantly reduce the number of M2-type macrophages in the tumor (Figure 5E). These results indicate that XA5508 can activate and increase the infiltration of CD4^+^ T cells and CD8^+^ T cells in the tumor, reduce the number of M2-type macrophages, and reverse the immunosuppressive TME in HCC.

To further verify the ability of XA5508 to alleviate the immunosuppressive TME, we conducted ELISA assays to examine the changes in cytokines in the serum of three groups of Hepal-6 orthotopic HCC mice (Model, cGAMP, XA5508). The results showed that compared to cGAMP, XA5508 could significantly increase the expression levels of immune factors IFN-β, IFN-γ, IL-2, IL-6, and TNFα in the mice (Figure 5F–H,J,K), and significantly reduce the expression of the immunosuppressive tumor-promoting factor IL-10 (Figure 5I). These results indicate that the liposome complex XA5508 activates the STING pathway through cGAMP, produces inflammatory factors, induces the polarization of M2-type macrophages in tumor tissues to M1-type, and reverses the immunosuppression.

Although the type I IFN released during STING activation is intended to amplify the immune response and attract other white blood cells, it also induces adaptive immune resistance in tumor cells, thereby weakening the response of tumor-specific T cells. Upregulation of immune suppressive factors such as PD-L1 is one of the main mechanisms of adaptive immune resistance [55]. We conducted an ELISA experiment to measure the changes in PD-L1 expression levels in tumor tissues of the Hepal-6 liver cancer model before the start of treatment (12 days after in situ cell inoculation) and after the end of treatment (27 days after in situ cell inoculation) (Figure S5). The results showed that after the end of treatment, the expression of PD-L1 in the tumor increased, and there was a significant difference compared to before the start of treatment. As an immunotherapy marker, the expression level of PD-L1 reflects the degree of tumor immune escape. Therefore, we quantitatively analyzed the expression levels of PD-L1 in the plasma and tumor cells of mice after the end of the treatment period (Figure 5L,M). The results showed that free cGAMP increased PD-L1 expression in serum and tumors, which was consistent with the results in the literature [55,56,57,58]. M1 macrophages activated by the cGAS-STING pathway can recruit T lymphocytes through the STING-IRF3 pathway, resulting in T lymphocyte infiltration in tumors [59]. The upregulation of PD-L1 increased the response to anti-PD-L1 Nb, thereby helping T cells recognize tumor cells, and after promoting the restoration of the normal immune response, the infiltrated T lymphocytes rapidly and effectively attacked tumor cells. After treatment with the complex XA5508, the expression level of PD-L1 in the tumor decreased, and there was a significant difference compared with the cGAMP group (Figure 5M). This result also explains well the advantages of cGAMP and anti-PD-L1 Nb in activating the immune system and blocking the immune escape strategy.

**Figure 6:**
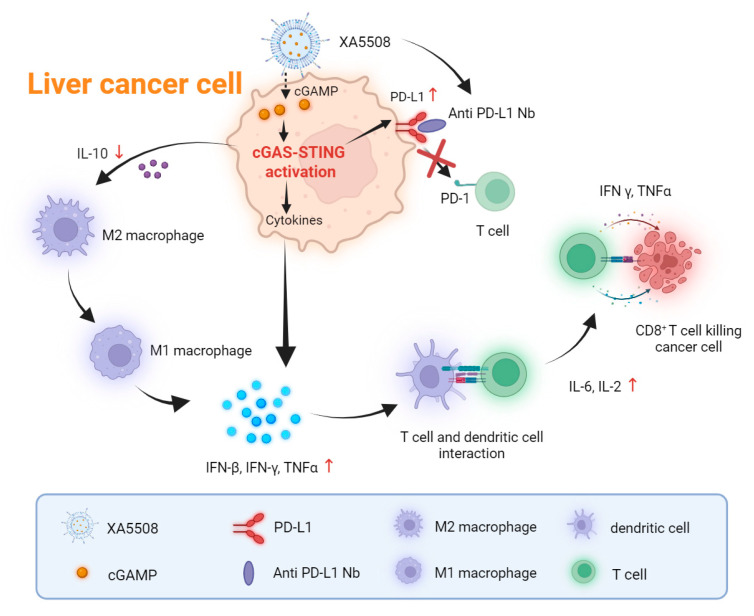
Schematic diagram illustrating the mechanism of action of liposome complex XA5508 treat liver cancer by both activating and enhancing the anti-tumor immune response and blocking immune escape.

Together, these results are summarized as shown in Figure 6. The anti-PD-L1 Nb coupled to the surface of liposomes achieves active tumor targeting, while the lipid nanocarriers are used to efficiently deliver cGAMP. In terms of mechanism, after XA5508 targets liver cancer cells, it releases cGAMP, activates the intracellular STING pathway, induces the production of immune cytokines, stimulates and activates CD8^+^ T cells, and simultaneously reverses the immunosuppressive TME characterized by M2-type macrophages, promoting the anti-tumor immune pathway to enhance the efficacy of anti-PD-L1 treatment. Additionally, cGAMP induces an increase in PD-L1 expression on the tumor cell surface, increasing the response rate of anti-PD-L1 Nb, thereby blocking the immune escape pathway of PD-1/PD-L1.

## 3. Discussion

In summary, we designed and prepared a liposome complex, XA5508, which encapsulated cGAMP and linked murine anti-PD-L1 Nb on the surface of liposome for immunotherapy of HCC. XA5508 utilized anti-PD-L1 Nb to recognize and bind to PD-L1 on the surface of tumor cells, sustainably delivered cGAMP into liver cancer cells, effectively and selectively activated the STING pathway in tumor cells, thereby enhancing the biological activity of cGAMP, stimulated the interferon response, and simultaneously reduced the immunosuppressive cell population in the TME, such as M2-type macrophages. cGAMP not only caused the upregulation of PD-L1 expression in tumor cells, but also facilitated the easier recognition of PD-L1 by anti-PD-L1 Nb and the blocking of immune escape caused by PD-L1.

Under normal circumstances, the activation of the STING signaling pathway leads to the production of IFN-I and inflammatory cytokines, thereby enhancing the anti-tumor immunity [60]. However, the activation of STING protein and antigen-presenting cells can also induce the expression of PD-L1 in tumor cells [16,58,61,62]. In line with these findings, our study observed an upregulation of PD-L1 following STING activation in our model, confirming that this is a conserved response (Figure 5). Blocking PD-L1 not only enables activated T cells to kill tumor cells expressing PD-L1, but also promotes the activation of dendritic cells to initiate T cells [63,64], thereby enhancing the sensitivity of tumor immunotherapy. Similar results have been observed in studies on the combination therapy of platinum-based chemotherapy and PD-1/PD-L1 inhibitors, where the combined therapy exerts a synergistic effect through two mechanisms [65]. On the one hand, the positive immunomodulatory effect of platinum-based chemotherapy can enhance the sensitivity of tumor cells to PD-1/PD-L1 inhibitors. On the other hand, platinum-based chemotherapy may upregulate the expression of PD-L1 in tumor tissues, exerting a negative immunomodulatory effect. And PD-1/PD-L1 inhibitors can counteract this effect through their action pathways.

There have always been considerable challenges in using the cGAS-STING pathway as a target for cancer treatment. Due to factors such as systemic toxicity, chronic immune activation, and tumor-promoting effects [66,67], the reported therapeutic efficacy of STING agonists in clinical trials has been limited. In particular, systemic administration of STING agonists may systemically activate STING, thereby inducing excessive pro-inflammatory factors to be secreted by both tumor and non-tumor tissues, and even triggering an immune storm [63,68], which has adverse effects on normal tissues and cells. Therefore, STING agonists administered systemically should specifically target tumor tissues and trigger anti-tumor immune responses in the TME, so as to maximize the anti-tumor effect and minimize the side effects on normal tissues. Due to the size characteristics of liposomes and the tail vein administration method, the liposomes flow from the hepatic portal vein blood to the liver and accumulate in the liver. Moreover, because the anti-PD-L1 Nb can selectively bind to tumor tissues with high PD-L1 expression, XA5508 can target both tumors and the liver. These dual characteristics provide excellent targeting for the treatment of liver cancer, which is an attractive research direction. Therefore, our research provides a new combined treatment strategy for HCC to enhance the sensitivity and response rate of liver cancer patients. Importantly, this immunoliposome strategy targeting Nb is superior to existing single-agent treatments or the combination of STING-NPs and ICB.

Although this study provided a comprehensive and multi-faceted description of how XA5508 selectively activates the STING signaling pathway, enhances anti-tumor immune responses, and inhibits immune escape to suppress the growth of liver cancer cells, it is still necessary to acknowledge some limitations. Firstly, researchers have currently developed many methods for establishing animal models of liver cancer. Although we conducted a pharmacological study on XA5508 using the Hepal-6 model and obtained beneficial results, it is important to note that some literature indicates that Hepal-6 can trigger a strong immune response and even lead to tumor regression. Therefore, it is difficult to fully represent the tumor immune microenvironment of the vast majority of human HCC cases. From this perspective, conducting preclinical pharmacological studies using this model is likely to affect the correct judgment of the effectiveness of future treatments for human HCC. Therefore, in the future, it is necessary to establish a liver cancer model that better conforms to the development characteristics and immune environment of human HCC to confirm the intervention and therapeutic effect of the liposome complex on liver cancer and its biological stability. Secondly, the analysis of PD-L1 was conducted based on the overall homogenate of the tumor, which limited the resolution at the cellular level and hindered the differentiation of molecular changes between non-tumor components (such as tumor cells, stromal cells, or immune cells). Therefore, to resolve the cell type-specific expression patterns, single-cell RNA sequencing or spatial transcriptomics is required. Another point to note is that the expression of PD-L1 in advanced liver cancer shows significant differences [10], which also leads to differences in the response of some patients to PD1/PD-L1 antibody therapy. Therefore, effectively treating these low immunogenic cold tumors remains a challenge. In our study, we observed that cGAMP activates the STING pathway and stimulates the secretion of type I interferons and pro-inflammatory cytokines. These factors can regulate the TME and convert cold tumors into hot tumors, thereby providing more significant benefits from immunotherapy [69]. Our findings provided a potential immunotherapeutic strategy for treating “cold tumors” that are difficult to treat by only administrating ICB therapy. Nonetheless, the mechanism of this combined strategy still requires further investigation to reveal its significance for clinical application. Additionally, the XA5508 selectively activates the STING pathway in tumor cells, triggering a strong systemic inflammatory response. This is necessary for enhancing the anti-tumor immune response, but its safety still requires continuous monitoring. Although these limitations highlight the direction for future research, they do not diminish the mechanistic and clinical relevance of our research results. We believe that this targeted strategy will provide better tumor control methods for further understanding of tumor immunotherapy. At the same time, targeted immunoliposomes may be a universal platform for delivering genes/drugs/chemicals to individual cancer cells simultaneously, targeting multiple signaling pathways.

## 4. Materials and Methods

### 4.1. Materials

2′,3′-cGAMP and the immunoliposome complex (XA5508) were provided by Hangzhou Orenstar Biomed Co., Ltd. (Hangzhou, China).; hydrogenated soybean phosphatidylcholine (HSPC, purity > 98%) and cholesterol (Chol, purity > 98%) were purchased from Aventis (Shanghai) Pharmaceutical Technology Co., Ltd. (Shanghai, China); 1,2-distearyl-san-glycer-3-phosphoethanolamine [methylene (polyethylene glycol)-2000] (mPEG2000-DSPE, purity > 98%) and DSPE-PEG2000-Mal were purchased from Xi’an Ruixi Biotechnology Co., Ltd. (Xi’an, China).

### 4.2. Preparation of Nanobody

Based on the literature [70], the Nb sequence was designed and the coding sequence was subcloned into the periplasmic expression vector pET-22b(+) of *Escherichia coli* (TransGen Biotech, Beijing, China) with 6 His tags at the C-terminal. The *E. coli* with the plasmid was cultured in LB medium containing ampicillin at 37 °C. After induction with 1 mM IPTG at 30 °C for 16 h, the cells were centrifuged and resuspended in buffer (50 mM Tris, 300 mM NaCl, pH = 8.0), and sonicated. It was then centrifuged at 10,000× *g* for 20 min at 4 °C and separated onto Ni-NTA buffer (50 mM Tris, 300 mM NaCl, 10 mM imidazole, pH = 8.0). The protein was eluted stepwise to 50 mM Tris, 300 mM NaCl, 500 mM imidazole, 10% glycerol, pH = 8.0. The eluted protein was loaded onto a Superdex S-200 column to improve purity. The recombinant Nb were identified by 15% SDS-PAGE and mass spectrometry. The purified protein was stored at −80 °C.

To remove bacterial endotoxins, the protein was immobilized on a 5 mL HisTrap HP column (GE Healthcare, Syracuse, NY, USA) in PBS. The protein was washed with PBS containing 0.1% Triton X-114, and then eluted with 500 mM imidazole in PBS without LPS. The imidazole was removed by dialysis. The LPS concentration was detected using the LAL colorimetric endotoxin quantification kit (Thermo Fisher Scientific, Waltham, MA, USA), and all proteins were purified of LPS (<2 IU/mg).

### 4.3. Liposomal Formulations Preparation

To prepare the nanoantibody-targeted liposome complex (XA5508), the Nb were modified with sulfhydryl groups according to the previous method [71]. The free sulfhydrylating reagent was removed by dialysis. The ammonium sulfate gradient method was used to prepare liposomes. To prepare fluorescent-labeled liposomes for in vivo fluorescence imaging, the near-infrared fluorescent probe DiR was loaded into the liposomes. In simple terms, the above lipids were dissolved in chloroform and evaporated to form a film at 40 °C. The ammonium sulfate solution was added to the phospholipid membrane and rotated to form multilayer liposomes. The liposomes were squeezed through a polycarbonate filter (Avastin) to obtain uniformly sized single-layer empty liposomes. After dialysis to remove the ammonium sulfate solution, the blank liposomes were obtained. The cGAMP solution was added to the blank liposomes, incubated at 60 °C for 1 h, and then dialyzed to remove the unencapsulated drugs. The sulfhydrylated nanoantibodies were added to the drug-loaded liposomes, incubated in the dark overnight, and then dialyzed to remove the free nanoantibodies.

### 4.4. Characterization of cGAMP-Loaded Liposomes

After mixing the demulsifier and liposomes, centrifuge at 12,000 rpm for 3 min to obtain the supernatant. Use high-performance liquid chromatography to establish a standard curve to determine the concentration of cGAMP. After high-speed centrifugation, inject the aqueous phase into an isothermal 20:80 (*v*/*v*) water/methanol mobile phase at a flow rate of 1.0 mL/min. After passing through the C18 column (Agilent, San Jose, CA, USA), measure the absorbance at 260 nm. The drug encapsulation rate (EE%) is calculated by the following formula:

$$\mathrm{EE}\%\;=\;\;\frac{{cGAMP}_{en}}{{cGAMP}_{tol}}\times 100\%$$

In the formula, cGAMP*_en_* represents the mass of the encapsulated cGAMP, and cGAMP*_tol_* represents the total mass of cGAMP.

The drug loading efficiency (LE%) is calculated by the following formula:

$$\mathrm{LE}\%\;=\;\frac{{cGAMP}_{mass}}{{liposome}_{mass}}\times 100\%$$

In the formula, cGAMP*_mass_* represents the mass of encapsulated cGAMP, and liposome*_mass_* represents the total mass of the loaded liposomes.

The average particle size distribution, polydispersity index (PDI), and zeta potential of the nano-targeted liposomes were determined by dynamic light scattering (Zetasizer Nano-ZS90, Malvern, UK). The morphology of the liposomes was observed using transmission electron microscopy (Tecnai G2 F20S-Twin, Hillsboro, OR, USA). The Nb connected to the surface of the liposomes was extracted by the methanol-chloroform oscillation extraction method. The concentration of the extracted protein was determined using the BCA kit. To evaluate the phospholipid concentration of the liposome complex, we used ultraviolet spectrophotometry. Take 25 μL of the liposome sample, add chloroform to 4 mL, then add 4 mL of the color development reagent. After shaking, centrifuge at 4000 rpm for 10 min, remove the upper layer, and measure the absorption of the lower layer at 485 nm using an ultraviolet spectrophotometer. Calculate the phospholipid concentration of the liposome sample based on the standard curve.

### 4.5. In Vitro Drug Release

Transfer the free cGAMP and XA5508 separately into dialysis bags (with MWCO of 3500 Da). Perform dialysis in 37 °C and 100 rpm conditions using PBS (pH 7.4) or PBS (pH 5.4). Take out 2 mL aliquots from the dialysis bags at predetermined time intervals and replace them with the same volume of fresh PBS. Determine the concentration of cGAMP using high-performance liquid chromatography and calculate its cumulative release rate using the formula:

$$\mathrm{Release}\;\mathrm{Percentage}\;=\;\frac{n_{i}C_{i}V+\sum n_{i-1}C_{i-1}V_{ex}}{W}\times 100\%$$

In the formula, *n_i_* represents the dilution ratio, *C_i_* is the concentration of each sample collected, V is the total volume of the dialysis medium, *V_ex_* is the volume of the solution extracted at each interval (2 mL), and W is the total amount of cGAMP.

### 4.6. Cell Lines

Hepal-6 cells were purchased from Hunan Fenghui Biotechnology (Changsha, China). Hepa1-6 cells were cultured in DMEM medium containing 10% fetal bovine serum (FBS) (Thermo Fisher Scientific, Waltham, MA, USA) and 1% P/S. The cells were incubated at 37 °C, 5% CO_2_, and 95% humidity.

### 4.7. Mice and Tumor Model Establishment

The Institutional Animal Care and Use Committee of Fudan University (Shanghai) authorized all the operations of this study by the animal experimentation ethics guidelines. The male C57BL/6J mice were purchased from Shanghai SLAC Laboratory Animal Co., Ltd. (Shanghai, China) and were housed in a pathogen-free animal facility. Unless otherwise specified, all the experimental animals were 6–8 weeks old and were housed in an environment-controlled breeding room with a temperature of 22 ± 2 °C and a relative humidity of 70% ± 5% (Certification No. SCXK(HU)2022-0004). Hepal-6 cells at a concentration of 1 × 10^6^ were injected subcutaneously into the right axilla of the C57BL/6J mice to establish a subcutaneous tumor model. The surgical operation for establishing the in situ liver cancer model is as described in reference [50]. Specifically, after shaving the skin, anisoflurane gas was used to anesthetize the mice. Alcohol cotton balls were used to wipe the abdomen. A cut approximately 1 cm long was made successively through the skin layer and peritoneal layer below the xiphoid process. The left liver lobe was gently compressed to extract it. Using an insulin needle, 2 × 10^6^ Hepa1-6 cells (tumor cells and matrix gel were mixed at a ratio of 1:1) were slowly injected 20 μL along the long axis of the liver. After the injection, the needle was slowly withdrawn. A sterile gel sponge was placed on the needle track to prevent leakage. The liver lobe was folded into the abdominal cavity, and the peritoneum and skin were gradually sutured. After disinfection with iodophor, the mice were placed on a warming pad until they regained consciousness. The tumor growth and survival conditions were observed every 2 days after inoculation.

### 4.8. In Vivo Tumor Inhibition Study

The mice carrying Hepal-6 were randomly divided into different treatment groups: Model group, cGAMP group, and XA5508 group. In this study, when the tumor volume reached 200 mm^3^, the mice were administered the drug every 2 days for a total of 7 times. The tumor volume was calculated as (L × W^2^)/2. L represents the maximum diameter of the tumor, and W represents the shorter vertical measurement of the tumor. To test the anti-tumor effect, the free cGAMP group and the XA5508 group were injected with the effective dose of cGAMP through the tail vein, which was 0.2 mg. We conducted a total of three independent and complete experiments. Data from one representative experiment out of 3 total performed. In each independent experiment, the number of samples in each group is *n* ≥ 3.

### 4.9. Hematoxylin and Eosin (H&E) Staining and Blood Chemistry Analysis

For H&E staining, the tumors were fixed with paraformaldehyde, embedded in paraffin, sectioned, and then stained with the H&E staining kit according to the manufacturer’s protocol (Sigma-Aldrich, St. Louis, MO, USA) using hematoxylin and eosin. To determine the toxicity of different treatments, whole blood and serum were collected, and blood routine and levels of alanine aminotransferase (ALT), total bilirubin (TBIL), creatinine (CREA), creatine kinase isoenzyme (CKMB), and lactate dehydrogenase 1 (LDH1) in serum were measured. Every trial conducted at least three times to ensure reliability. Each data point represents one animal (biological replicate). Groups consisted of *n* = 5 independent animals.

### 4.10. Flow Cytometry Analysis of Immune Cells in the Tumor Tissues

Tumor tissues were isolated from mice, and the tumor fragments were ground in RPMI-1640 medium through a 70 μm filter to prepare a single-cell suspension. The cell suspension was centrifuged, and red blood cells were removed using erythrocyte lysis buffer. After two washes, M2-TAMs were labeled as CD45^+^CD11b^+^F4/80^+^CD206^+^. CD8^+^ T cells were labeled as CD3^+^CD8^+^. CD4^+^ T cells were labeled as CD3^+^CD4^+^. All antibodies were purchased from BioLegend(San Diego, CA, USA) and detected using a flow cytometer (Agilent NovoCyte, Santa Clara, CA, USA). We conducted a total of three independent and complete experiments. Data from one representative experiment out of 3 total performed.

### 4.11. Enzyme-Linked Immunosorbent Assay

The mouse blood samples were centrifuged at 3000 rpm for 20 min. The concentrations of IFN-β, IFN-γ, IL-6, IL-10, IL-2, TGFβ and PD-L1 in the plasma were detected using the mouse ELISA kit (FANKEW, Shanghai, China). The mouse tumor tissues were respectively added with an appropriate amount of physiological saline and made into 10% homogenate solutions under an ice water bath. These solutions were then centrifuged at 3000 rpm for 20 min. The contents of PD-L1 in the tumors and plasma were detected using the mouse ELISA kit. We conducted a total of three independent and complete experiments. Data from one representative experiment out of 3 total performed.

### 4.12. In Vivo Imaging

Approximately 2 × 10^5^ Hepal-6 cells were inoculated in situ into the liver of male C57BL/6J mice. Two weeks later, when the tumor volume reached approximately 500 mm^3^, the mice were randomly divided into 3 groups. The mice were injected with XA5508 (DiR), Lip-empty (DiR), and anti-PD-L1 Nb (DiR) via the tail vein 48 h later. The mice were then sacrificed, and their hearts, livers, spleens, lungs, kidneys, and tumors were removed to obtain in vitro fluorescence images. The average fluorescence intensity of the images was analyzed using Living Image 4.0 software. Each data point represents one animal (biological replicate). Groups consisted of *n* = 5 independent animals.

### 4.13. Immunofluorescence

The tumor tissues were washed with pre-cooled PBS (pH 7.4) and then fixed in 4% paraformaldehyde for more than 24 h. After gradient ethanol dehydration, they were embedded in paraffin. Then, the tissues were cut into 5 μm thick sections, and the sections were baked at approximately 50 °C. The dried tissue sections were placed in xylene for decolorization, followed by gradient ethanol hydration. The sections were subjected to antigen retrieval, blocked with 5% BSA for 30 min, and washed with PBS (pH 7.4). The tissue sections were then treated with 0.1% Triton X-100 in PBS (pH 7.4) for 5 min and washed with PBS (pH 7.4). Diluted STING antibodies (Abcam, Cambridgeshire, UK, 1:200, *v*/*v*), p-STING antibodies (Abcam, 1:200, *v*/*v*), and IRF3 antibodies (Abcam Cambridgeshire, UK, 1:200, *v*/*v*) were added for staining of the target proteins and incubated at 4 °C overnight. The sections were washed with PBS (pH 7.4). Fluorescent secondary antibodies (Abcam, 1:200, *v*/*v*) were added in the dark for 2 h at room temperature and then washed with PBS (pH 7.4). Finally, the tissue sections were stained with DAPI (Beyotime, Shanghai, China) in the dark for 10 min and then washed with PBS. After the tissue dried, they were sealed with neutral resin and observed and photographed under a fluorescence inverted microscope. Each data point represents one animal (biological replicate). Groups consisted of *n* = 3 independent animals.

### 4.14. Statistical Analysis

Data are expressed as bars, and line graphs and presented as mean ± SEM. The *n* indicates the number of animals per experimental group, as reported in the figure and figure legends. The statistical significance of the difference for a single comparison was evaluated with an unpaired Student’s *t* test. One-way or two-way ANOVA with Turkey’s test and Benjamini–Hochberg correlation were used for multiple-group comparisons limited to three planned pairwise comparisons. For all analyses, group differences were considered statistically significant when * *p* < 0.05, ** *p* < 0.01, *** *p* < 0.001, and **** *p* < 0.0001. The analysis used GraphPad Prism version 9.00 for Windows and Macintosh, GraphPad Software 9.3.1 (471), La Jolla, CA, USA. Details of statistical tests are provided in each of the legends for the main and Supplementary Figures S1–S5 and Supplementary Table S1.

## 5. Conclusions

In this study, we developed and characterized a novel lipid nanocomplex, XA5508, aiming to overcome the bottlenecks of low immunogenicity and immune escape in the effective immunotherapy of HCC. Our research results demonstrated that by connecting anti-PD-L1 nanobodies to the surface of liposomes carrying cGAMP, cGAMP could be specifically targeted and delivered to hepatoma cells expressing PD-L1. This would specifically activate the STING signaling pathway in tumor cells, trigger robust interferon response, and reshape the TME. Through systematic evaluation in an in situ Hepal-6 liver cancer model, it was found that XA5508 could significantly inhibit tumor progression. Based on the experimental results, we inferred a reasonable pharmacological mechanism: cGAMP-mediated STING activation could significantly upregulate the expression of PD-L1 in tumors. This phenomenon is usually considered to be the cause of drug resistance and immune suppression, but here it enhanced the targeting efficiency and immune-blocking effect of our nanoplatform, forming a synergistic cycle of innate immune activation and adaptive immune enhancement.

Our research provides two important insights: (1) Nanocarriers can enhance targeting and immunogenicity by optimizing the immune feedback loop (STING → IFN → PD-L1 upregulation), converting an anti-tumor mechanism into a therapeutic advantage. (2) Simultaneously delivering a STING agonist and immune checkpoint blocker spatially to the same cell is a superior strategy for achieving synergistic anti-tumor immunity. These advancements in knowledge offer a new perspective for combined immunotherapy of HCC and other related diseases.

To sum up, XA5508 exhibited superior tumor-targeting capability, potent antitumor efficacy, and effective activation of the STING-mediated immune response in an orthotopic HCC model. The key quantitative findings from these experiments are summarized in Table 4.

## Figures and Tables

**Table 1:** Characterization results of liposome complex XA5508.

	XA5508
Particle size (nm)	202.06 ± 3.81
Zeta potential (mV)	−14.30 ± 1.30
PDI	0.182 ± 0.08
EE (%)	83.06 ± 2.68
LE (%)	8.31 ± 0.17
Protein concentration (mg/mL)	256.40 ± 33.49
Phospholipid concentration (mg/mL)	12.57 ± 0.45

The data from 3 repetitions were analyzed using mean ± SD. EE: Encapsulation efficiency. LE: Drug loading efficiency.

**Table 2:** Blood routine indicators were detected after the end of the administration cycle.

	Normal Reference Range	Normal	Model	Model + XA5508
WBC	0.8–10.6 (10^9^/L)	6.8 ± 1.2	3.3 ± 0.1	12.7 ± 1.8
Gran	0.23–3.6 (10^9^/L)	2.4 ± 0.6	1.7 ± 0.2	9.5 ± 1.6
RBC	6.5–11.5 (10^12^/L)	8.4 ± 1.9	8.0 ± 1.3	7.3 ± 1.3
HCT	35–55 %	44.2 ± 1.8	33.8 ± 2.7	34.5 ± 3.7
PLT	400–1600 (10^9^/L)	1030.0 ± 134.0	720.5 ± 58.7	1107.5 ± 64.3

**Table 3:** Represent liver, kidney and heart function indicators were detected after the end of the administration cycle.

	Normal Reference Range	Normal	Model	Model + XA5508
ALT	10.1–96.5 (U/L)	31.6 ± 3.9	29.0 ± 4.6	33.7 ± 7.8
TBIL	6.1–53.1 (μmol/L)	37.5 ± 2.4	35.1 ± 4.2	42.7 ± 4.7
CREA	10.9–85.1 (μmol/L)	32.7 ± 3.7	24.3 ± 4.8	27.6 ± 4.5
CKMB	0–2070.6 (U/L)	220.5 ± 30.3	140.0 ± 20.1	150.8 ± 39.6
LDH1	0–37.1 (U/L)	14.8 ± 5.2	15.3 ± 1.0	17.6 ± 1.5

**Table 4:** Summary of main results for XA5508.

Key Findings	Highlight Results	Figures to Display the Results
Tumor-targeted delivery	Achieved effective targeted delivery of cGAMP to tumor tissues.	Figure 2A,B
pH-dependent drug release	Effectively released cGAMP under acidic conditions simulating the tumor microenvironment.	Figure 2C
In vivo therapeutic efficacy	The tumor suppression rate of in situ HCC reached 86% after 2 weeks of treatment.	Figure 3F
STING pathway activation	The expression levels of STING protein, phosphorylated STING protein, IRF3 protein, and pro-inflammatory cytokines increased.	Figure 4 and Figure 5F–H,J,K
Enhance biological effects of innate immune activation while blocking immune escape	The infiltration of CD4^+^ and CD8^+^T cells in the tumor tissue increased. XA5508 inhibits the upregulation of PD-L1 in tumor tissues caused by cGAMP.	Figure 5C,D,M

## Data Availability

Original data can be obtained from the authors upon reasonable request.

## Supplementary Materials

The following supporting information can be downloaded at: https://www.mdpi.com/article/10.3390/ijms26178649/s1.

## Abbreviations

The following abbreviations are used in this manuscript:

HCC	Hepatocellular carcinoma
ICB	immune checkpoint blockade
TME	Tumor microenvironment
PD-1	programmed cell death protein-1
PD-L1	programmed death ligand 1
Nb	Nanobody
