# Supplementary material for: A Study on the Efficacy and Pharmacological Mechanism of Liposome Complexes Containing STING Agonist and Anti-PD-L1 Nanobody in Inhibiting HCC
Source: Int J Mol Sci. 2025 Sep 5;26(17):8649. doi: 10.3390/ijms26178649 (PMC12429036; doi:10.3390/ijms26178649)
Supplement: Supplementary file 1 [file ijms-26-08649-s001.zip › ijms-3846758-supplementary.pdf]

Supplementary information

for

A Study On the Efficacy and Pharmacological Mechanism of Liposome Complexes Containing STING Agonist and Anti-PD-L1 Nanobody in Inhibiting HCC

Xiaoqing Wang , Xing Lu , Chang Liu , Hao Cheng and Xiangshi Tan *

*Correspondence and requests for materials should be addressed to:
xstan@fudan.edu.cn

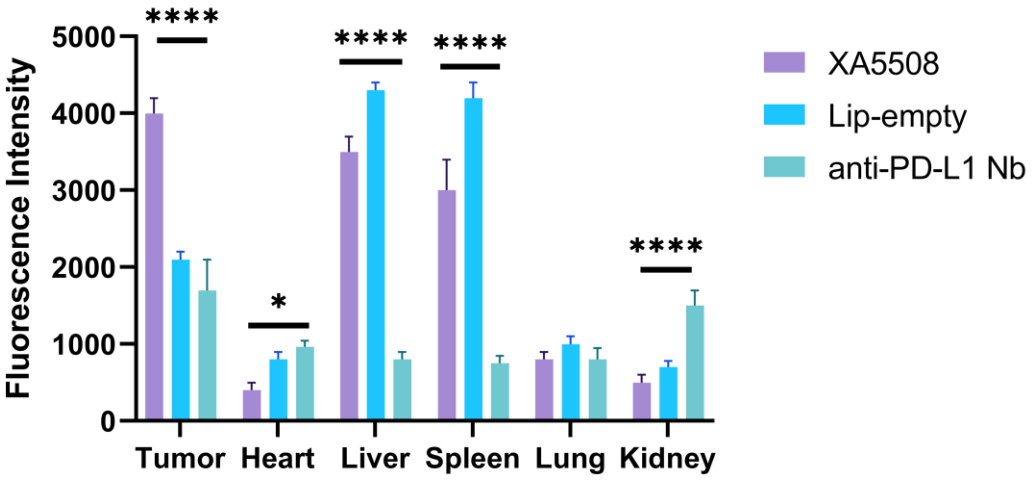

Supplementary Figure S1. Statistical analysis of the fluorescence intensity of the in vitro tissues of tumor-bearing mice 48 hours after injection of DiR liposomes. n=5, Data are means±SEM. Two-way ANOVA with Turkey's test and Benjamini–Hochberg correlation were used for multiple-group comparisons limited to three planned pairwise comparisons. *, $p < 0.05$; ****, $p < 0.0001$.

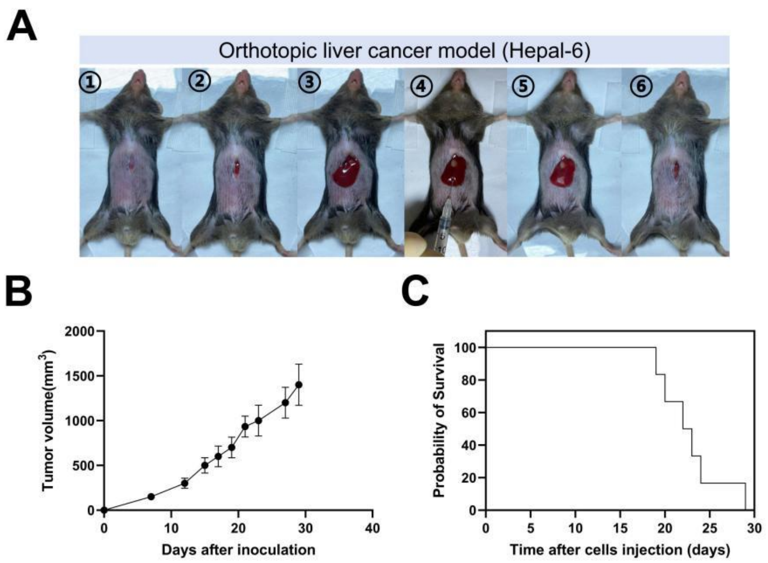

Supplementary Figure S2. We established a mouse in situ liver cancer model. (A) The modeling method and surgical operation process of the Hepa1-6 mouse in situ liver cancer model. (B) The tumor growth curve of the Hepa1-6 in situ liver cancer model. (C) The survival curve of the Hepa1-6 in situ liver cancer mice. n = 5. Data are means ± SEM. One-way ANOVA with Tukey's HSD significant test was used for pairwise comparisons.

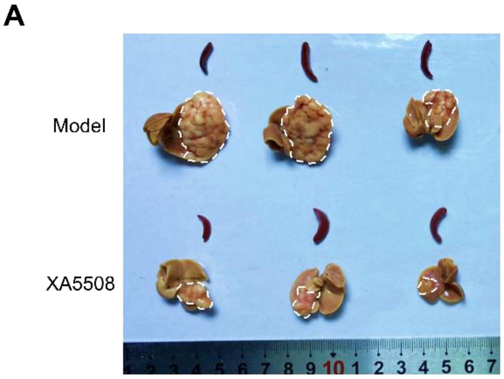

B

Group	Average tumor volume (mm³)	Tumor inhibition rate (%)
model	1977.62	/
XA5508	216.53	89

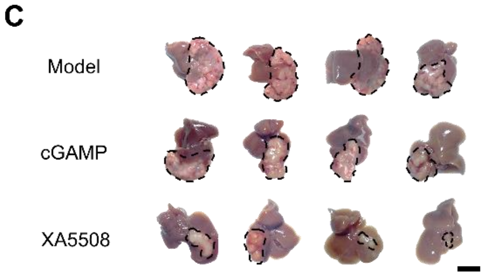

D

Group	Average tumor volume (mm³)	Tumor inhibition rate (%)
model	1833.62	/
cGAMP	992.00	45
XA5508	229.63	87

Supplementary Figure S3. Photos of tumors and tumor inhibition rates from two independent repeated experiments in the in vivo efficacy test of XA5508. (A, B) Photos of tumors and tumor inhibition rates from one independent repeated experiment. (C, D) Photos of tumors and tumor inhibition rates from one independent repeated experiment.

Supplementary Table S1. Blood routine test results for XA5508 administration after 24 hours and 1 week.

	Normal reference range	Normal	Normal+XA5508 (24h)	Normal+XA5508 (1 week)
WBC	0.8-10.6 (10^9/L)	6.8±1.2	8.9±0.7	6.6±2.1
Gran	0.23-3.6 (10^9/L)	2.4±0.6	3.1±0.8	2.1±1.3
RBC	6.5-11.5 (10^12/L)	8.4±1.9	7.5±1.4	7.6±1.5
HCT	35-55 %	44.2±1.8	40.6±3.5	38.4±2.9
PLT	400-1600 (10^9/L)	1030.0±134.0	1280.3±77.1	960.7±100.5

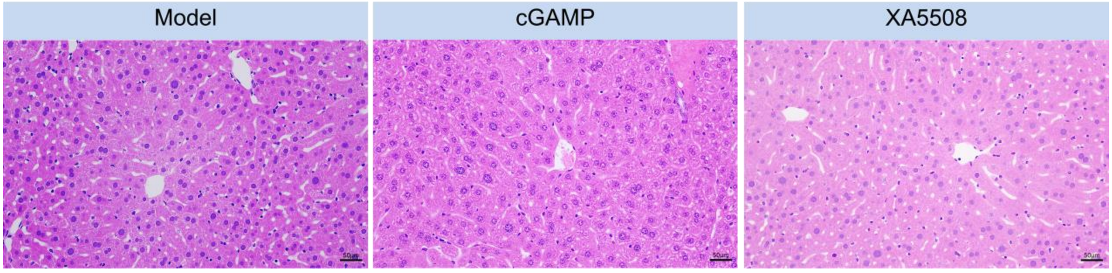

Supplementary Figure S4. HE staining of non-tumor liver tissues after the completion of the Hepal-6 in situ liver cancer experiment.

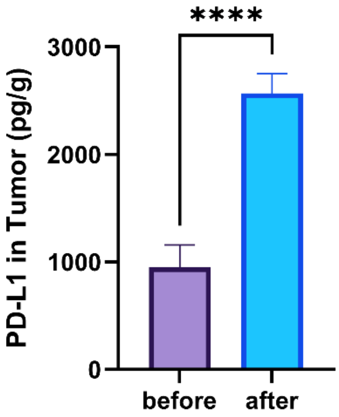

Supplementary Figure S5. The changes in PD-L1 expression levels in tumor tissues of the Hepal-6 liver cancer model before the start of treatment (12 days after in situ inoculation of cells) and after the end of treatment (27 days after in situ inoculation of cells). *n*=4. The comparison of means between the two groups was conducted using the unpaired t-test. ****, $p < 0.0001$ vs before the start of treatment.
